# Supplementary material for: A synthetic microbial biosensor for high-throughput screening of lactam biocatalysts
Source: Nat Commun. 2018 Nov 29;9:5053. doi: 10.1038/s41467-018-07488-0 (PMC6265244; doi:10.1038/s41467-018-07488-0)
Supplement: Supplementary file 1 — Supplementary Information [file 41467_2018_7488_MOESM1_ESM.pdf]

A synthetic microbial biosensor for high-throughput screening of lactam biocatalysts

Yeom *et al.*

a

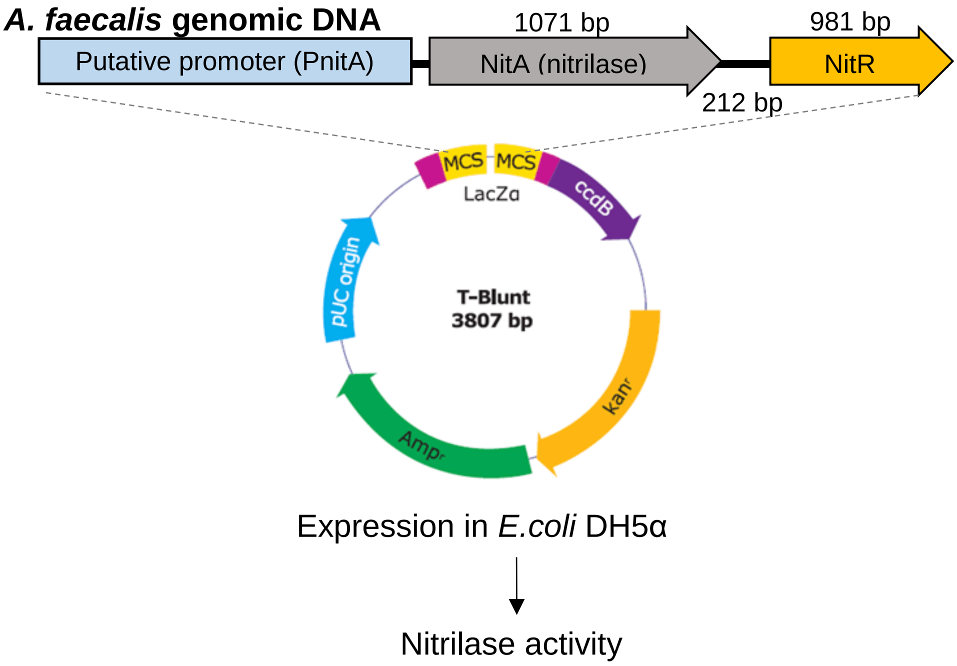

b

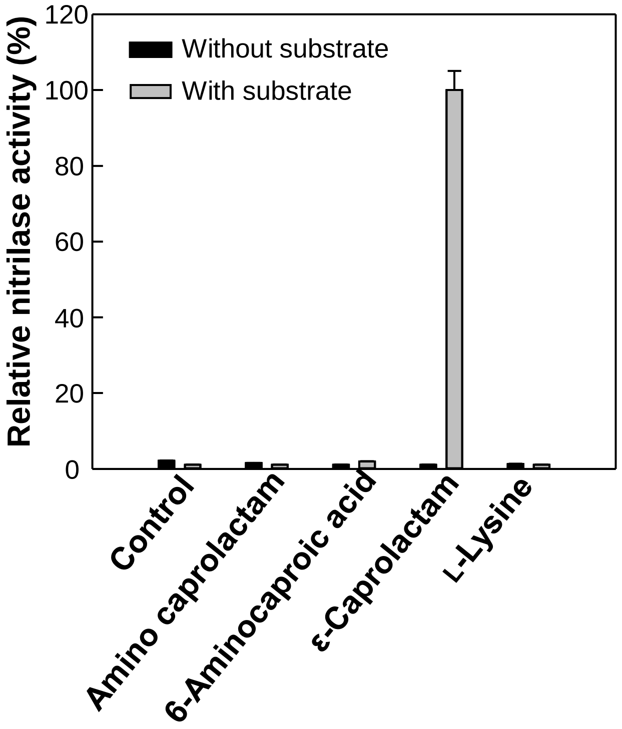

Supplementary Fig.1. Induced nitrilase activity by addition of ε-caprolactam. a. Cloning and expression of whole nitrile degrading part from *A. faecalis* genomic DNA. **b**. Analysis of nitrilase activity under control of NitR in presence of various ligands. Values represent means ± SDs of three independent experiments.

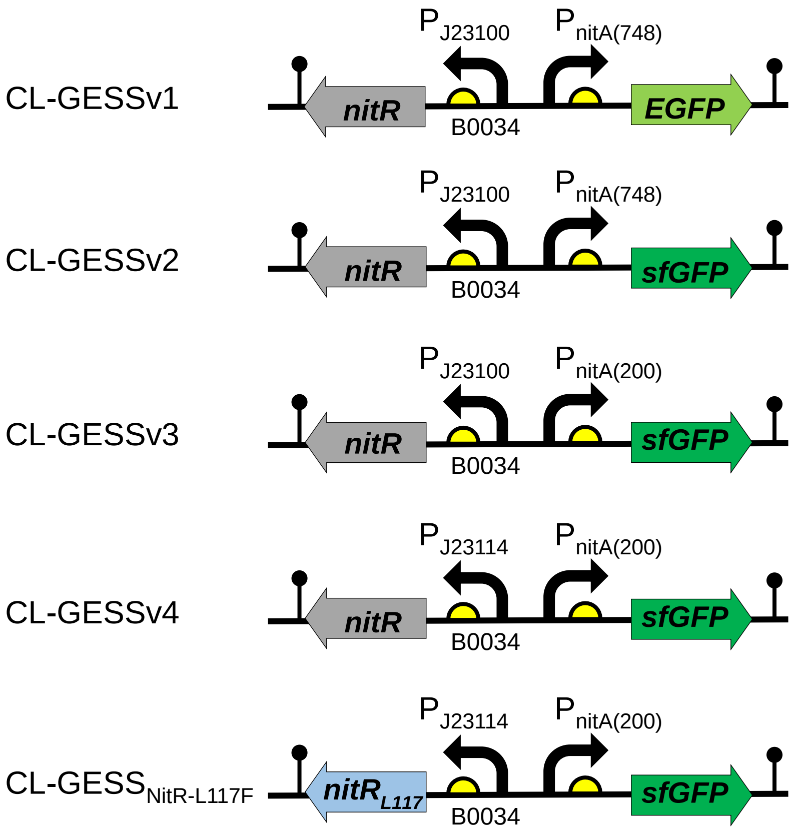

Supplementary Fig.2. Different versions of CL-GESS constructs. Plasmid CL-GESSv1 contains the codon-optimized *NitR* under the control of constitutive promoter J23100. *NitR* regulates the promoter $P_{nitA(748)}$. CL-GESSv2 contains sfGFP instead of EGFP. CL-GESSv3 has the truncated promoter $P_{nitA(200)}$. CL-GESSv4 has a weaker promoter J23100. CL-GESS$_{NitR\text{-}L117F}$ contains a mutant NitR and showed the highest sensitivity toward ε-caprolactam in this study.

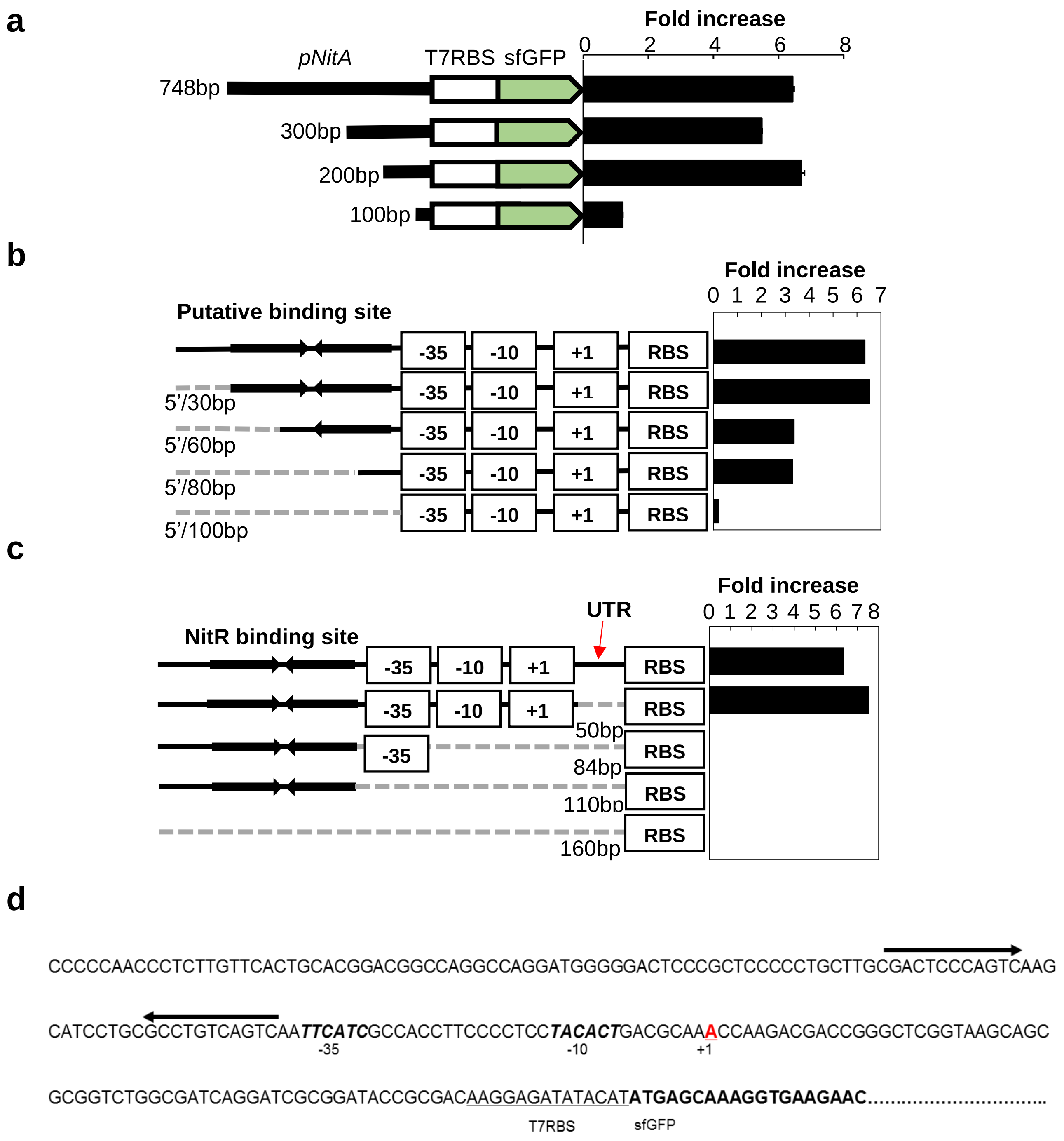

Supplementary Fig.3. Optimization of *A. faecalis nitA* promoter region for CL-GESS biosensor. a. Truncation of *nitA* promoter. **b.** Determination of the TF-binding site. **c.** Determination of Shine-Dalgarno sequences by sequence truncation. **d.** Nucleotide sequence of the region flanking the *A. faecalis nitA* promoter. Shine-Dalgarno sequences are underlined. The red adenine residue is the transcription start site of nitA determined by primer extension. Palindromic sites are indicated by converging arrows. Values represent means ± SDs of three independent experiments.

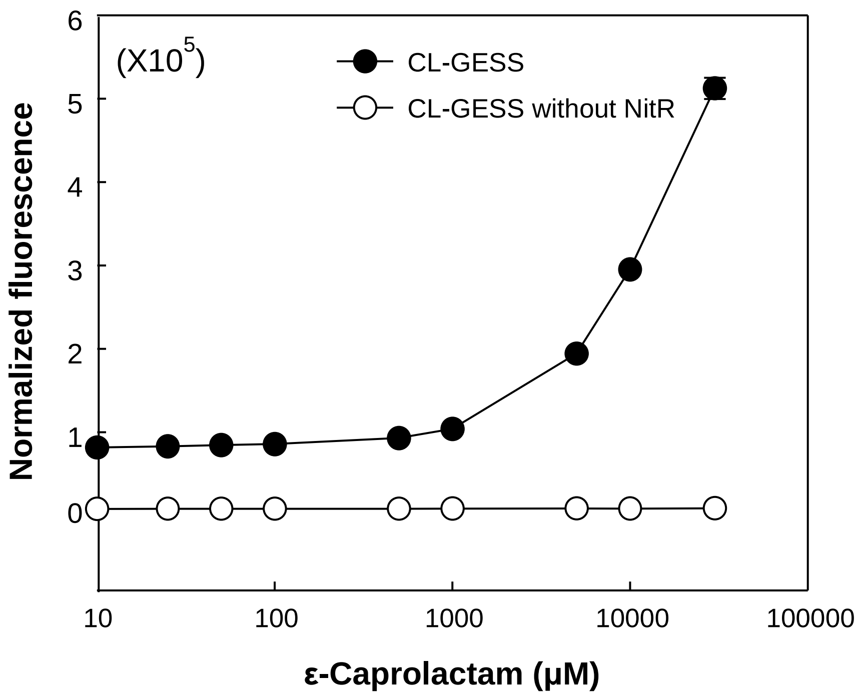

Supplementary Fig.4. Validation of NitR regulator function dependent on ε-caprolactam concentrations. Cells harboring the CL-GESS (closed circle) and those without NitR (open circle) were cultivated in LB medium at various concentrations of ε-caprolactam for 16 h.

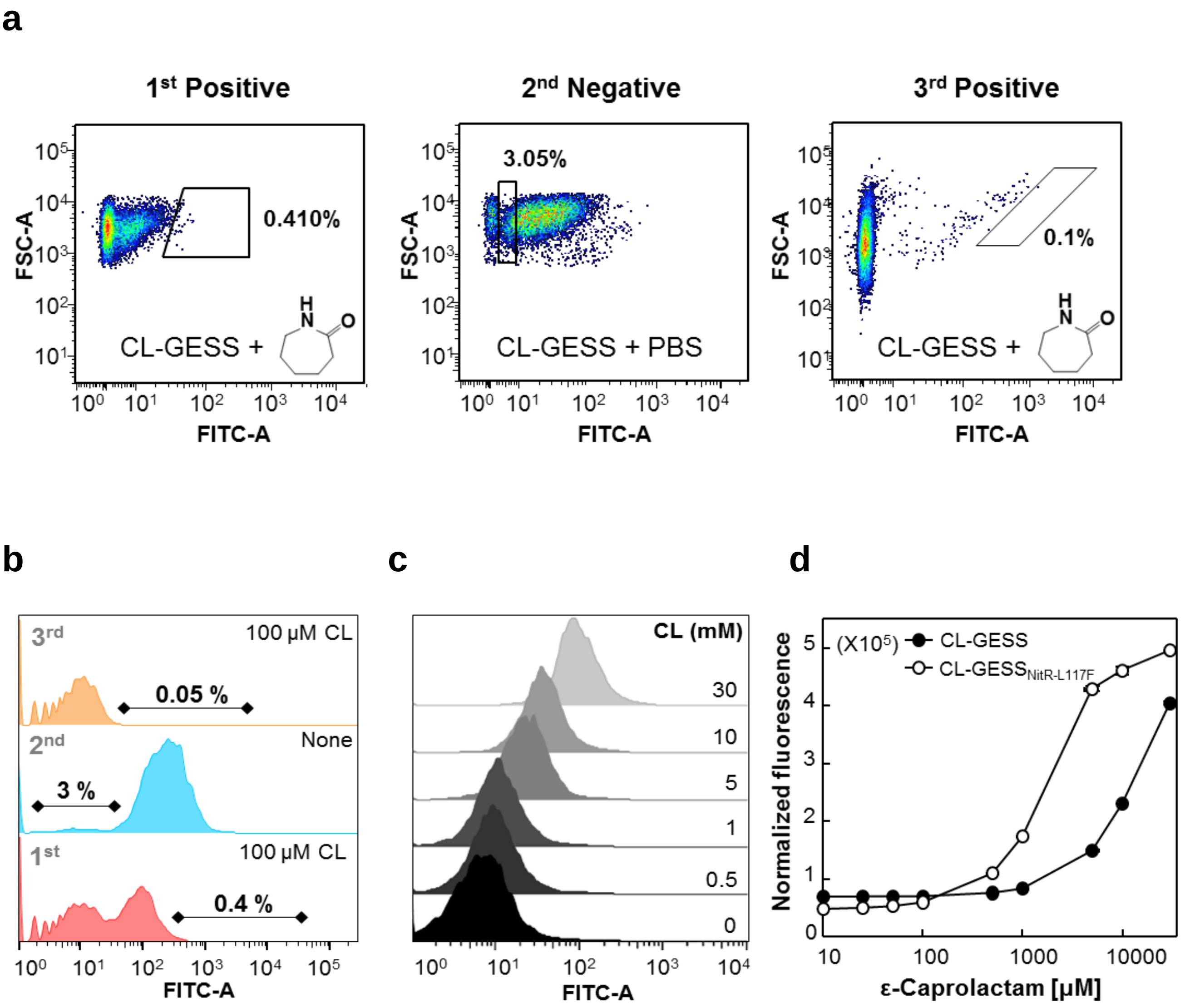

Supplementary Fig.5. Directed evolution of NitR regulator. a. Consecutive FACS-based screenings. NitR library was subjected to three stages of library screenings: 1st, sorting of positive clones for higher fluorescence intensity; 2nd: removal of false-positive cells showing fluorescence in the absence of ε-caprolactam; 3rd, enrichment of strongly fluorescent cells by the presence of ε-caprolactam. Lined boxes are the windows to recover the corresponding cells. **b.** Fluorescence histograms of the same library. **c**. CL-GESS profiles of NitR-L117F. **d**. Fluorescence intensities of wild-type and L117F NitRs. Normalized fluorescence was compared at different ε-caprolactam concentrations. Values represent mean ± SD of three independent experiments.

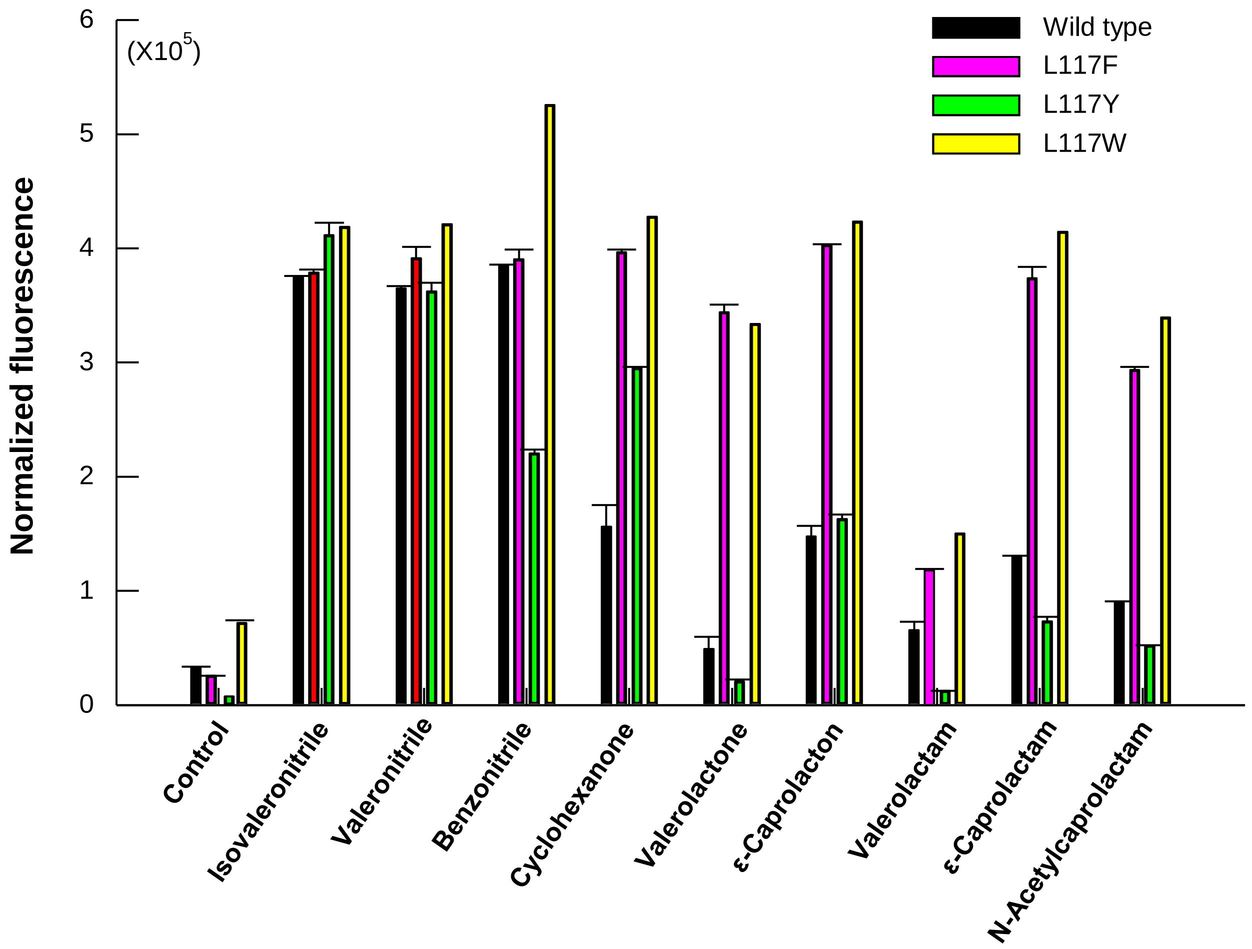

Supplementary Fig.6. Ligand specificities of CL-GESS$_{NitR-L117F}$,CL-GESS$_{NitR-L117Y}$, CL-GESS$_{NitR-L117W}$ and wild type CL-GESS. Various ligands (1 mM) were added to LB medium containing CL-GESS or mutant CL-GESS cells. Values represent the mean ± SD of three independent experiments.

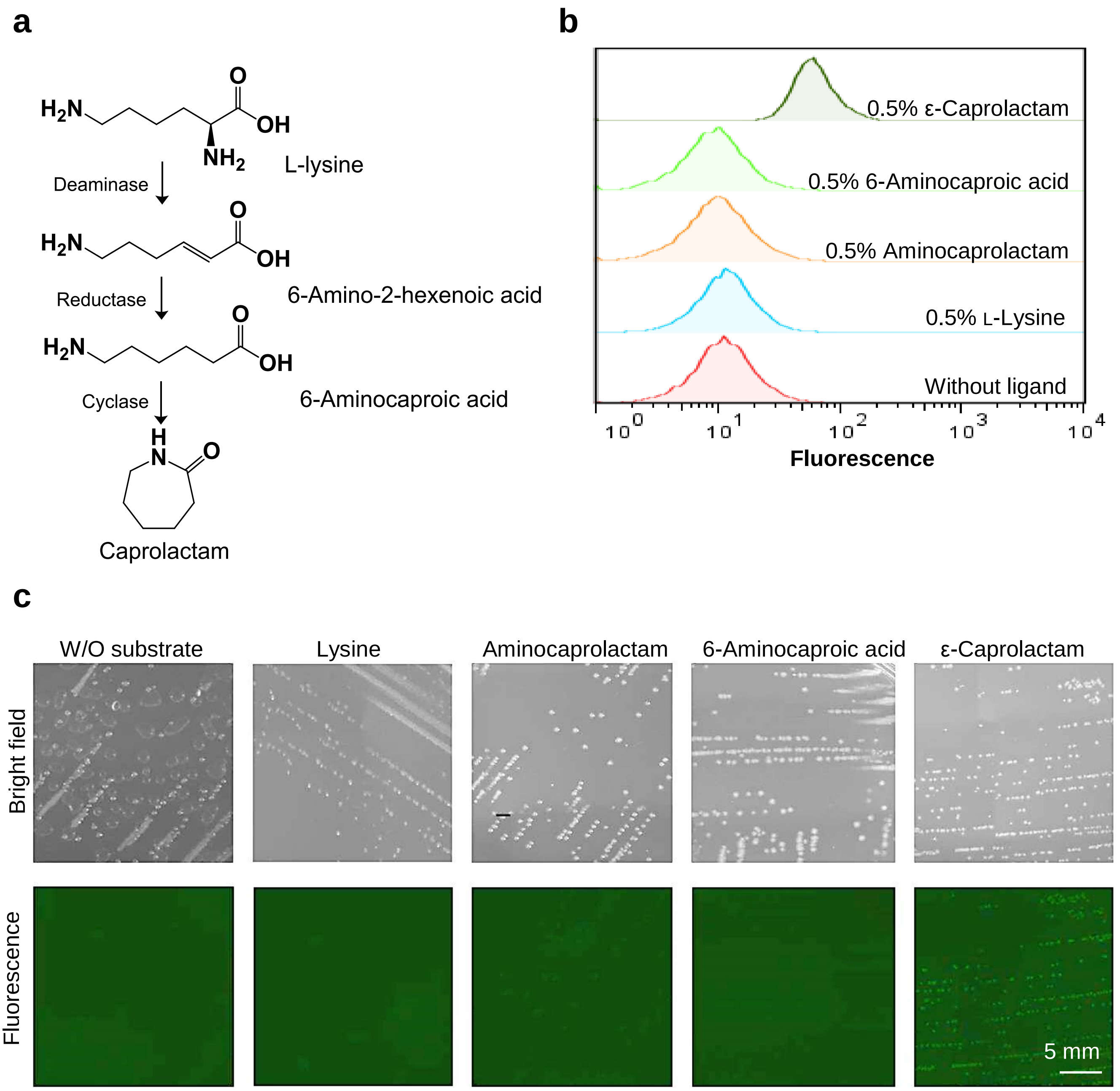

Supplementary Fig.7. Examination of various precursors in ε-caprolactam synthetic pathway using CL-GESS cells. a. Proposed synthetic pathway of ε-caprolactam from L-lysine as starting biochemical. **b**. Single-cell analysis by FACS in presence of various substrates. **c.** Fluorescence test of various precursors using CL-GESS on LB plate. All precursors with 10-50 mM for ε-caprolactam biosynthesis did not show fluorescence.

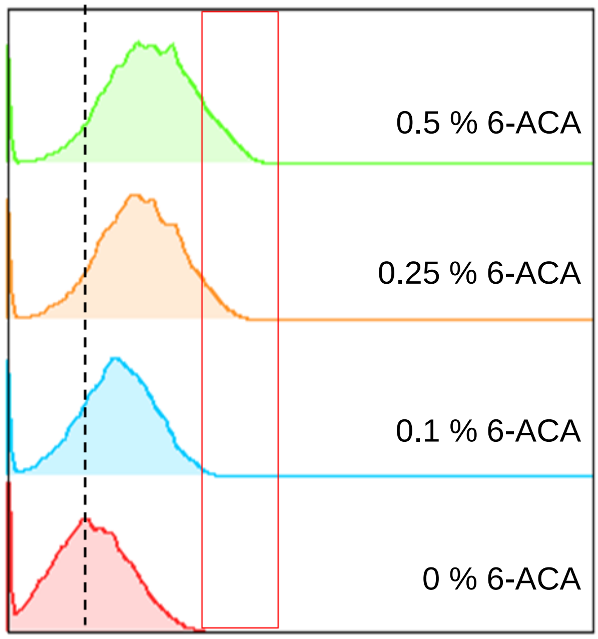

Supplementary Fig.8. Flow cytometric analysis of #3 fosmid hit with CL-GESS toward various concentrations of 6-aminocaproic acid.

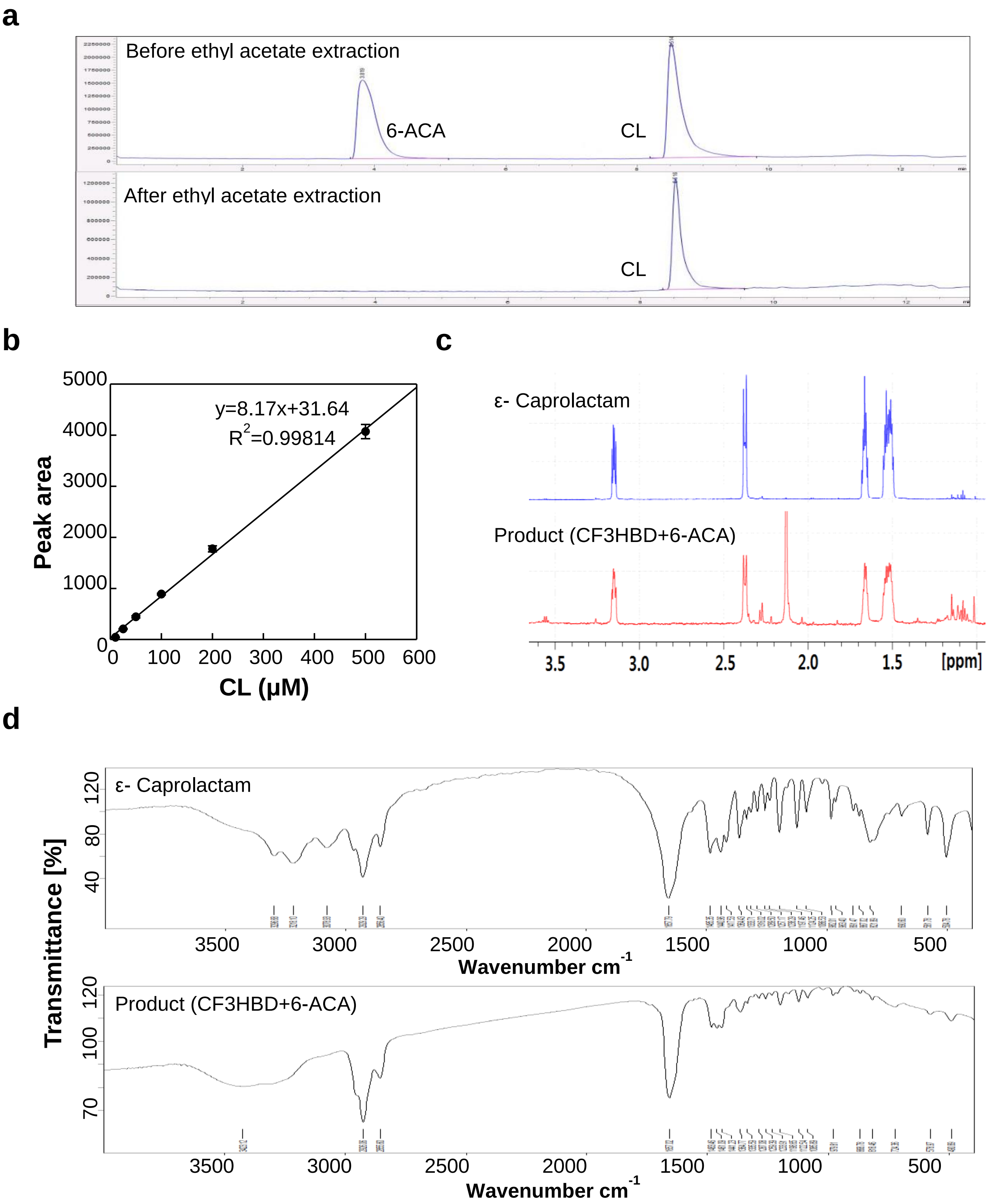

Supplementary Fig.9. **Identification of ε-caprolactam by LC-MS, NMR, and IR. a.** LC-MS analysis of the CF3HBD reaction product before and after the ethyl acetate extraction. **b.** CL-peak areas proportional to the concentrations of authentic ε-caprolactam. Y-axis indicates the ion intensity of the ESI positive ion mode (m/z 50–200) for LC–MS. n=5. **c.** 700 MHz 1H NMR spectra of the authentic ε-Caprolactam (upper) and the purified product from the reaction mixture (lower). **d**. IR spectra of the authentic ε-caprolactam and the purified product.

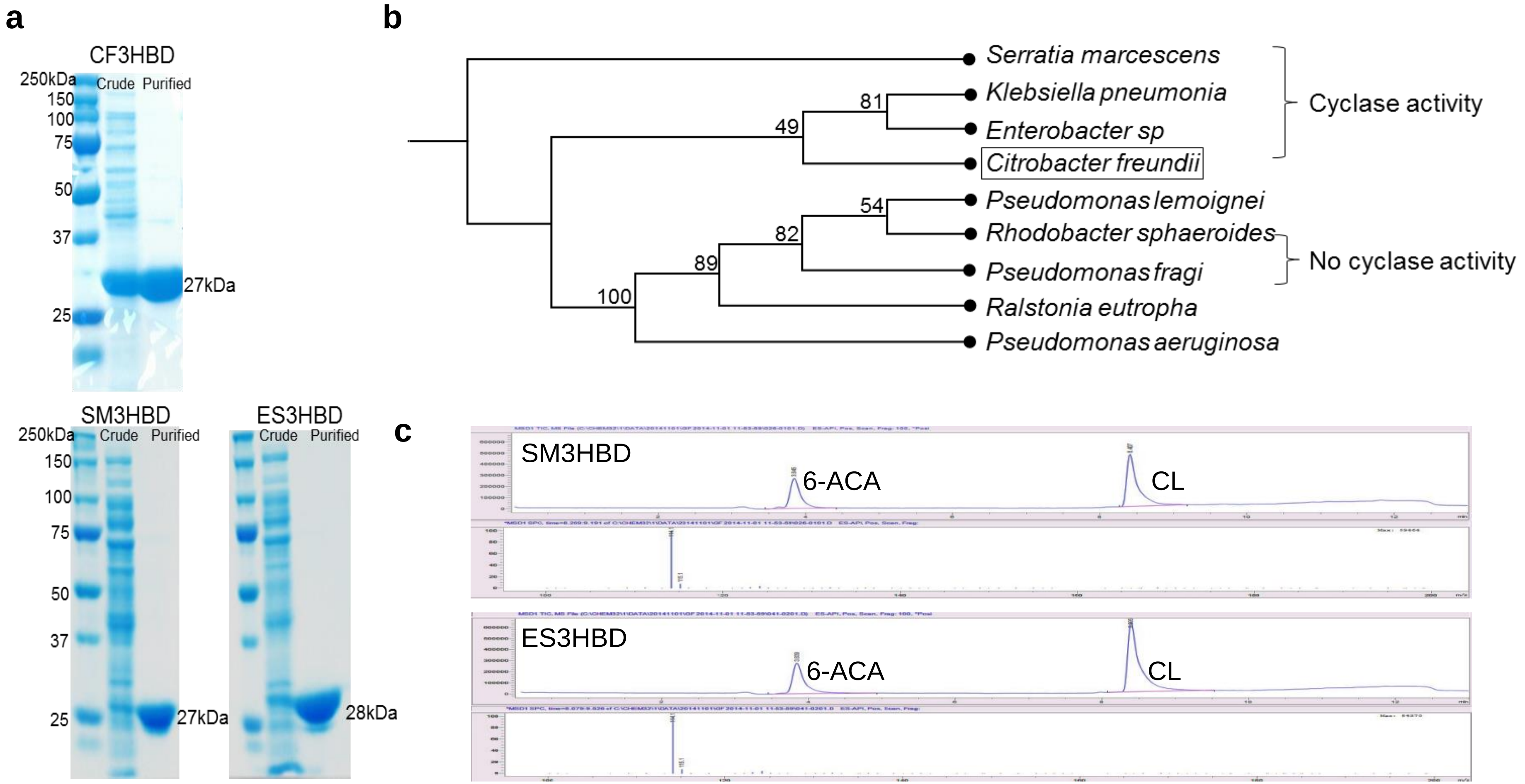

Supplementary Fig.10. Distribution of the cyclase activities among phylogenetic homologs. a. Expression and purification of tested homologs using sodium dodecyl sulfate-polyacrylamide gel electrophoresis analyses. **b.** Phylogenetic relations of 3HBD from various species based on sequence similarity. *Serratia marcescens*, WP_033651391.1; *Citrobacter freundii*, WP_003843689.1; *Klebsiella pneumonia*, WP_032435148.1; *Enterobacter* sp, WP_032657847.1; *Pseudomonas aeruginosa*, WP_023103221.1; *Ralstonia eutropha*, AAD33952.1; *Pseudomonas fragi*, WP_049071820.1; *Rhodobacter sphaeroides*, 2.4.1: WP_017140204.1; *Pseudomonas lemoignei*, BAF81903.1. **c**. LC-MS analysis of reaction mixture for SM3HBD and ES3HBD in the presence of 6-aminocaproic acid. The reaction was carried out using 0.5 mg/ml purified enzyme and 1 mM 6-aminocaproic acid as the substrate at 35°C and 50 mM HEPES buffer (pH 7.5).

a

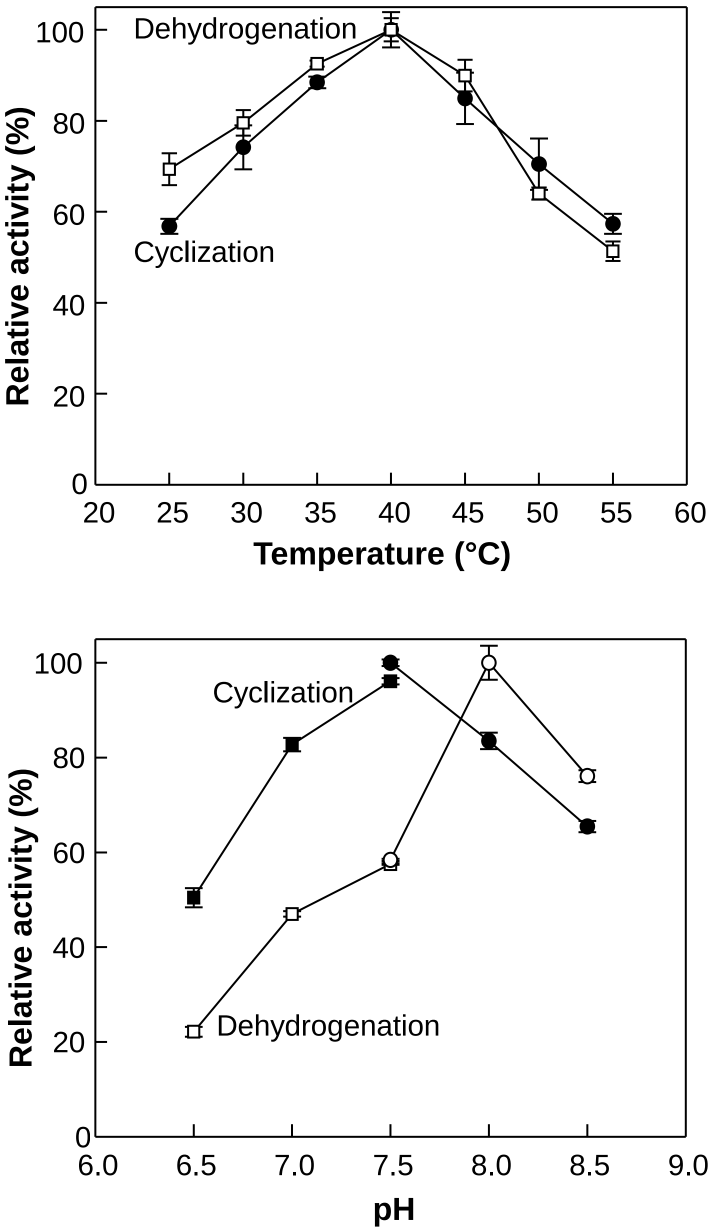

b

Supplementary Fig.11. Effects of pH and temperature on dehydrogenation (red symbol) and cyclization activities (black symbol) of 3HBD. **a.** Temperature: Dehydrogenation and cyclization reactions were performed in 50 mM HEPES buffer (pH 8.0) with 5 mM 3-hydroxybutyrate and 50 mM HEPES buffer (pH 7.5) with 1 mM 6-aminocaproic acid for 10 min, respectively. **b**. pH: Dehydrogenation and cyclization reactions were performed in 50 mM PIPES (square) or HEPES (closed circle) containing 5 mM 3-hydroxybutyrate and 1 mM 6-aminocaproic acid, at 40ºC for 10 min, respectively. Values represent means ± SDs of three independent experiments.

a

b

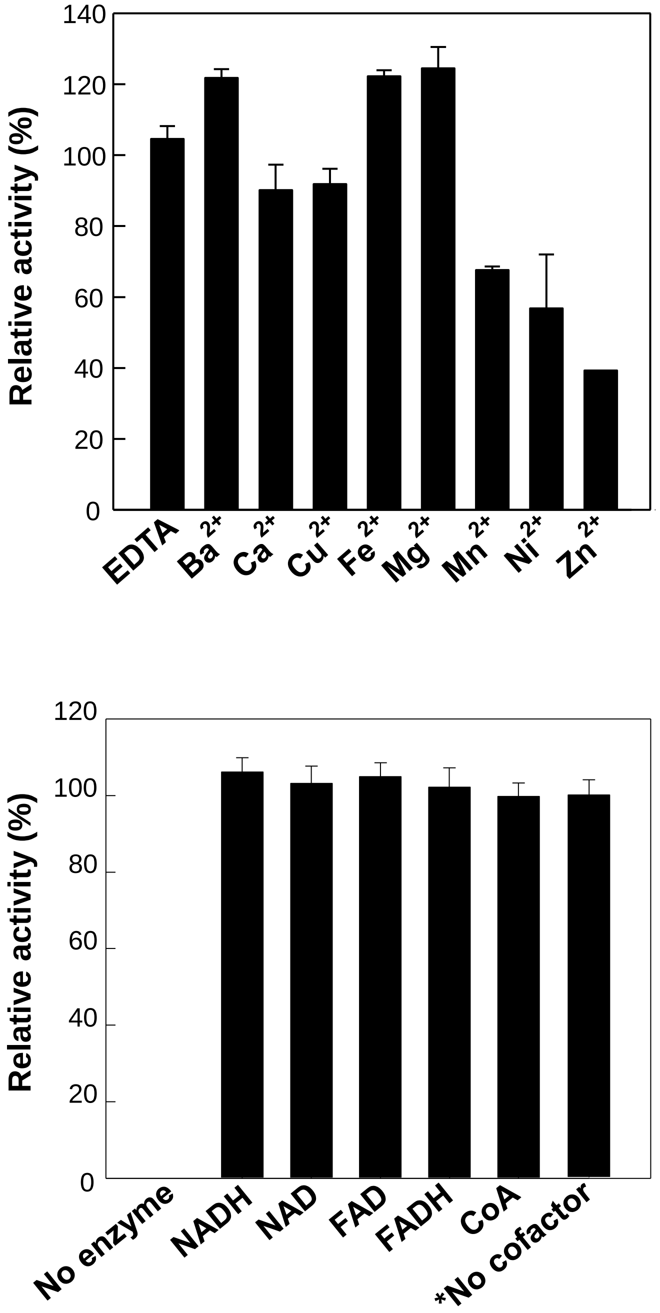

Supplementary Fig.12. Effects of metals and cofactors on 6-aminocaproic acid cyclization. a. Effects of metal ions. The reactions were performed in 50 mM HEPES buffer (pH 7.5) with 1 mM 6-aminocaproic acid, purified CF3HBD, and 1 mM of divalent ions at 40ºC for 10 min. **b.** Effects of cofactors. The reactions were performed in 50 mM HEPES buffer (pH 7.5) with 1 mM 6-aminocaproic acid, purified CF3HBD, and 10 mM of cofactors at 40ºC for 10 min. Error bars represent standard deviations of three experiments.

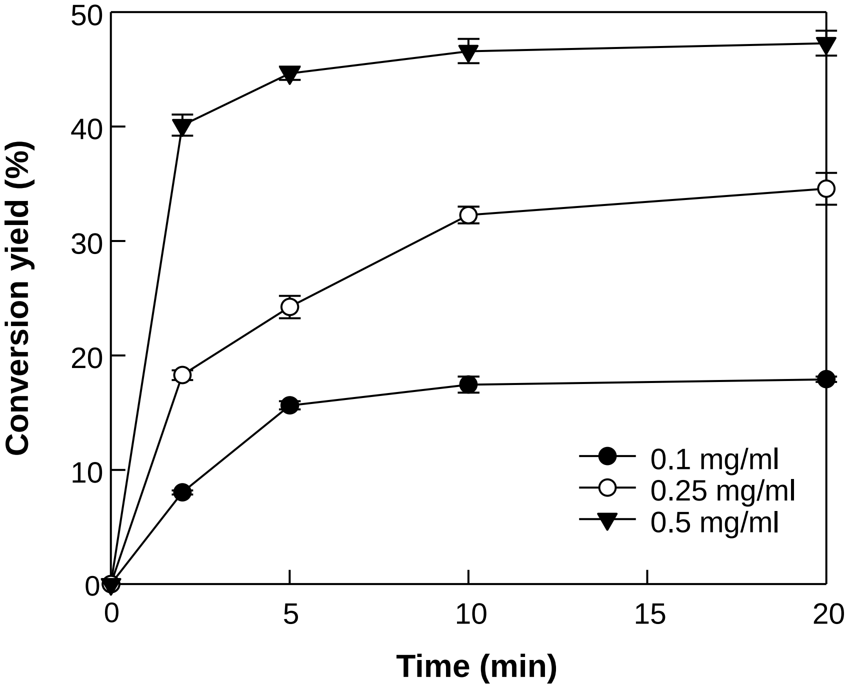

Supplementary Fig.13. Bioconversion of ε-caprolactam production from 6-aminocaproic acid by CFHBD. The reactions were performed in 50 mM HEPES buffer (pH 7.5) with 1 mM 6-aminocaproic acid at 40ºC with various concentration of purified CF3HBD. Values represent means ± SDs of three independent experiments.

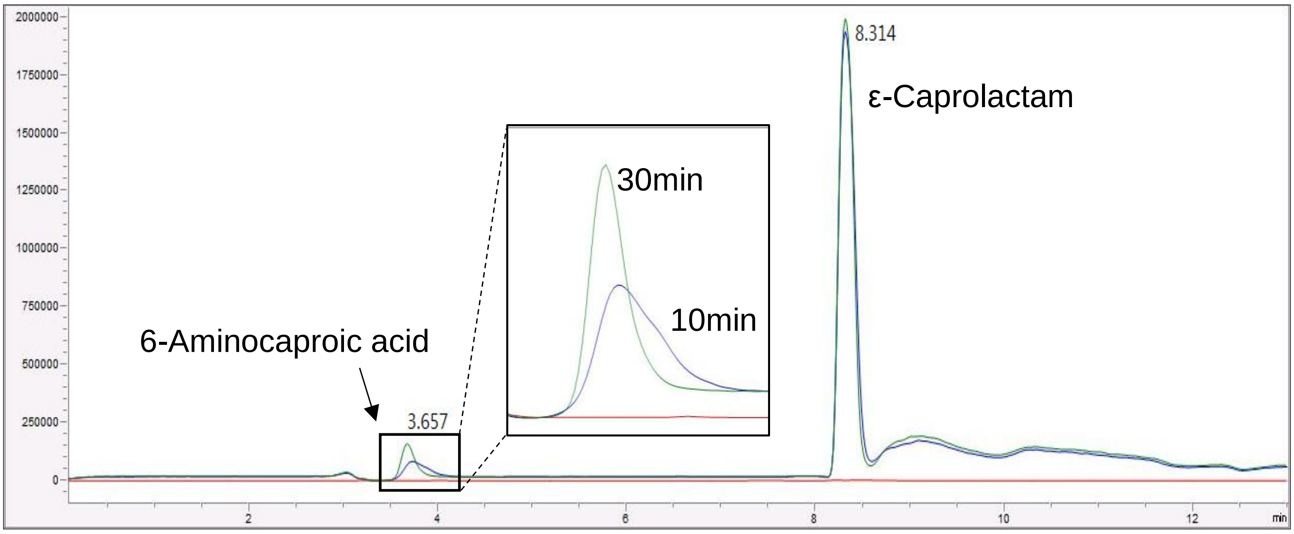

Supplementary Fig.14. ε-Caprolactam ring-opening amidase activity of purified CF3HBD. The increasing peak of 6-aminocaproic acid shows that CF3HBD has a ring-opening amidase activity. The reactions were performed in 50 mM HEPES buffer (pH 7.5) with 1 mM ε-caprolactam at 40ºC with CF3HBD at 10 and 30 min.

a

b

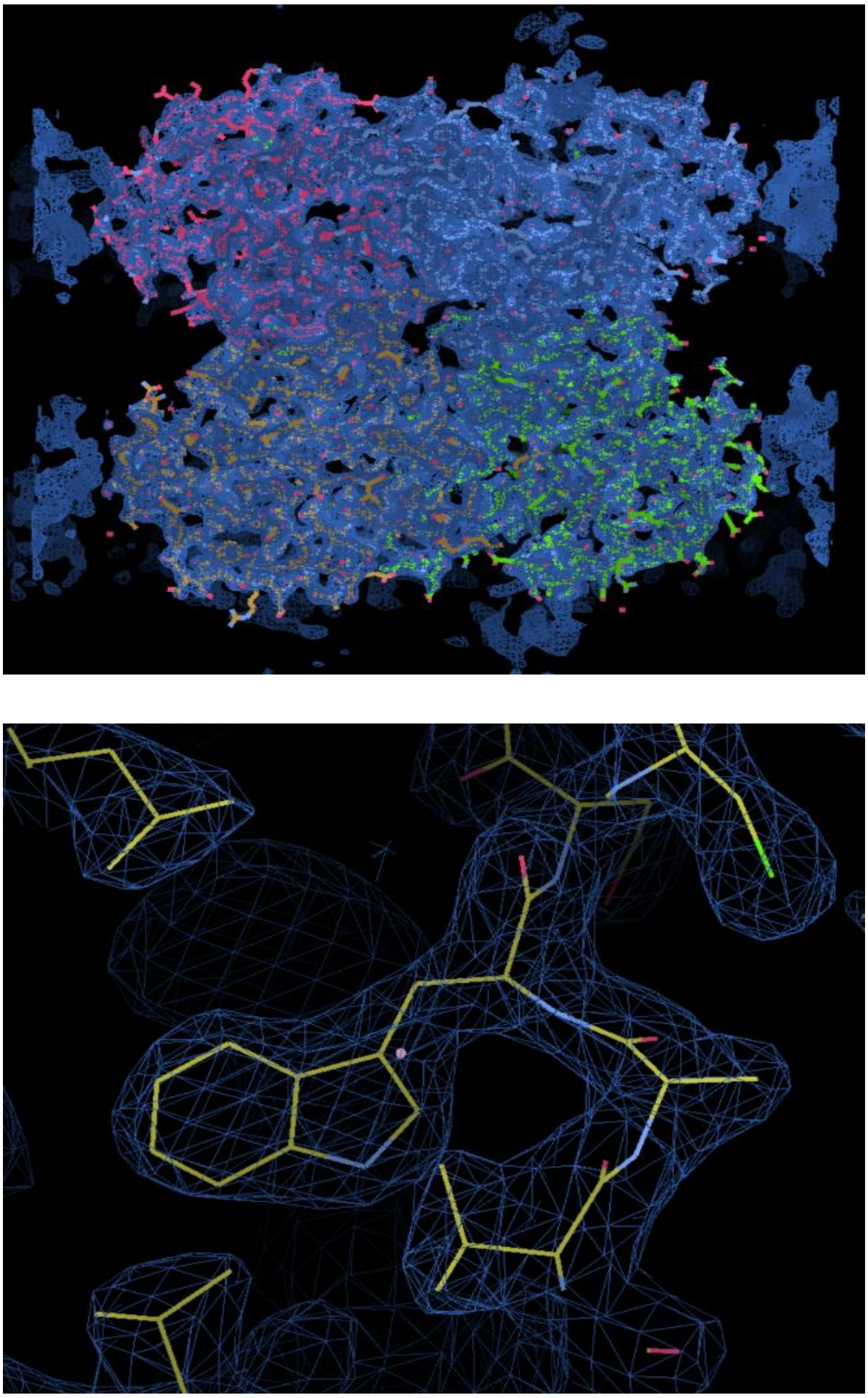

Supplementary Fig.15. The electron density map of CF3HBD. The figures were generated by *WinCoot*. The $2F_0$-$F_c$map contoured at 1.5σ is shown as blue mesh. The refined atomic model of CF3HBD are shown as line with different color scheme by atoms; yellow-carbon, red-oxygen, blue-nitrogen, and green-sulfur. No crystallographic symmetry intimates the tetrameric structure of CF3HBD (**a**). The map represents the high resolution of 2.4 Å of CF3HBD (**b**).

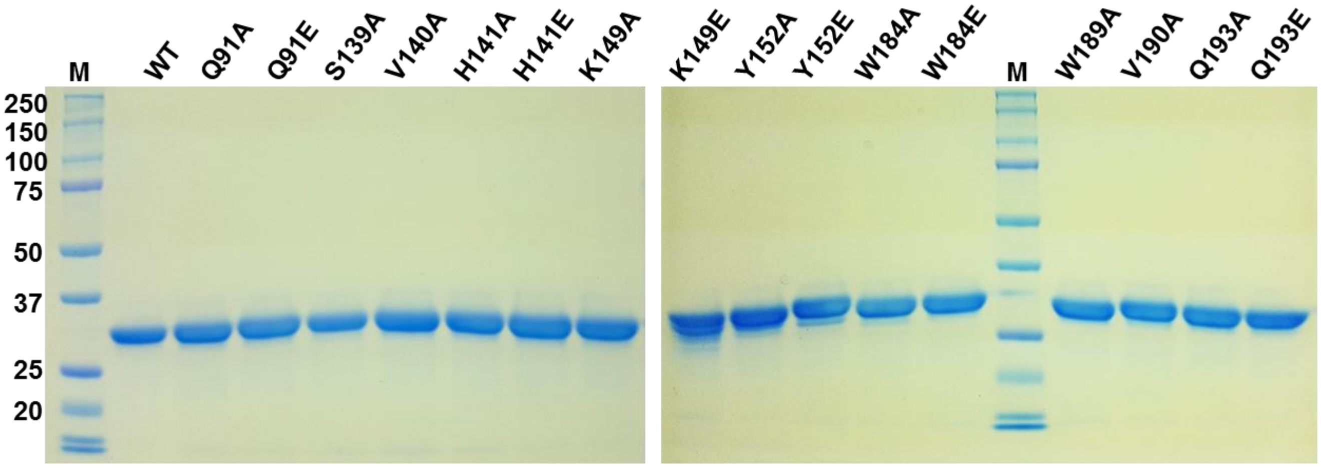

Supplementary Fig.16. Sodium dodecyl sulfate-polyacrylamide gel electrophoresis of purified 3HBD and various site-directed mutants. M, prestained marker proteins.

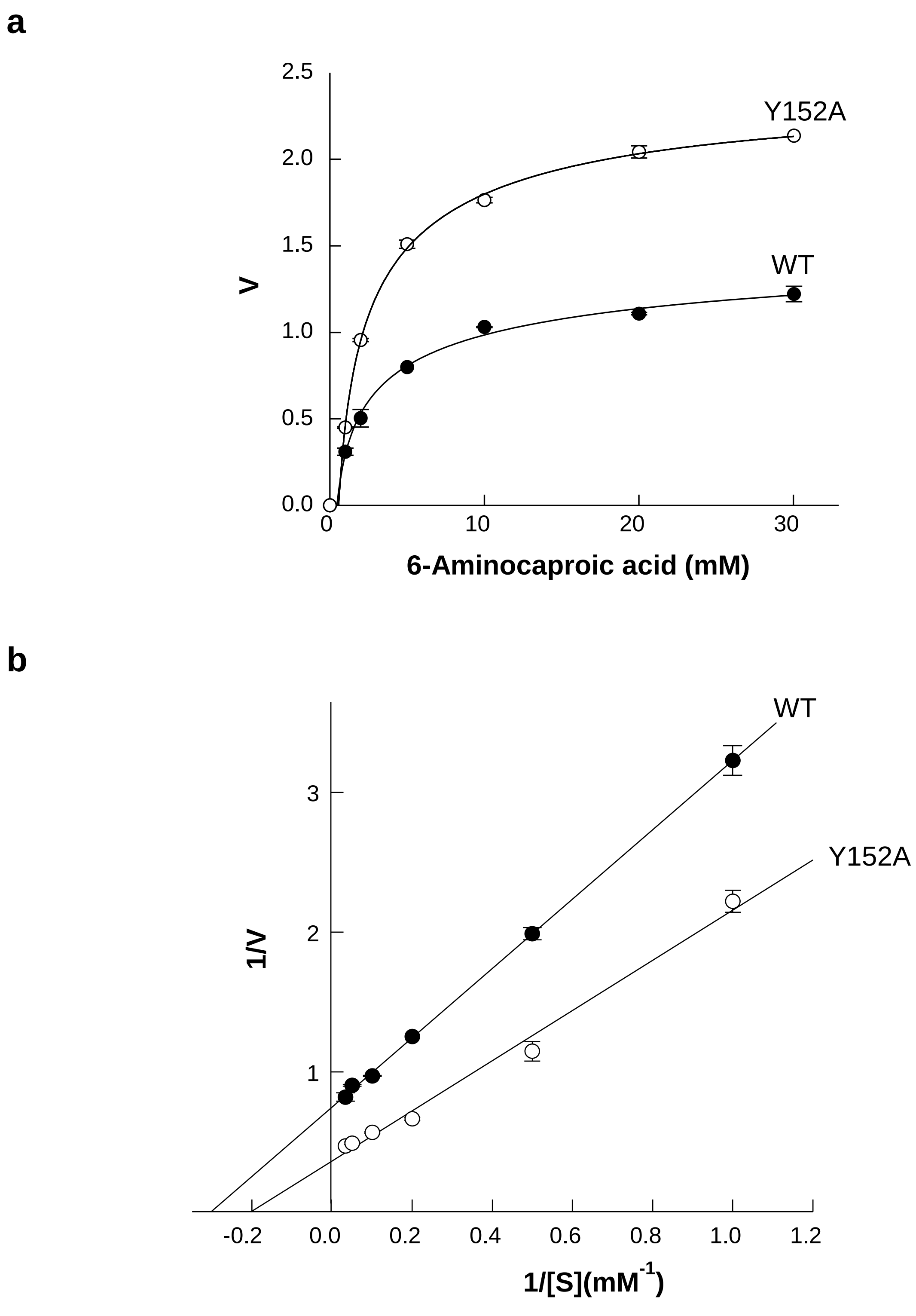

Supplementary Fig.17. Kinetic analyses of the 3HBD and Y152A mutant. a. Michaelis–Menten plot of 3HBD wild type (closed symbol) and Y152A mutant (opened symbol) using 6-aminocaproic acid as the substrate. **b.** Lineweaver-Burk analyses of the same data. The reaction was performed in 50 mM HEPES buffer (pH7.5) at 40 °C for 10 min. Values represent means ± SDs of three independent experiments.

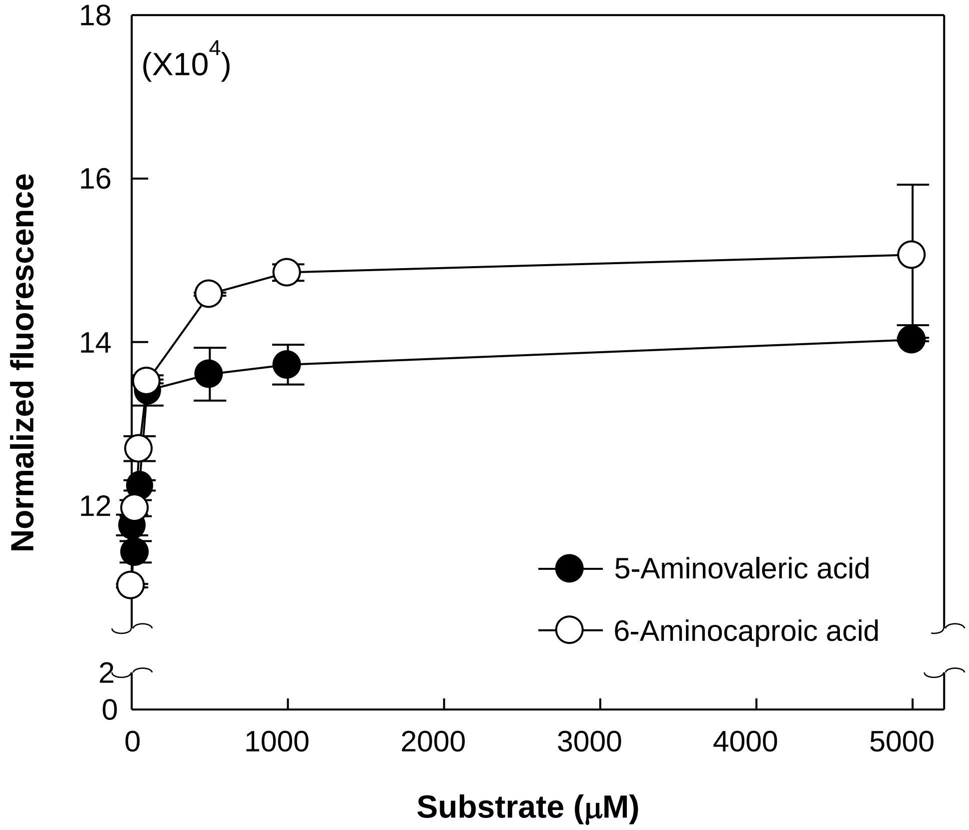

Supplementary Fig.18. Detection of 5-aminovaleric acid and 6-aminocaproic acid by the combination of CL-GESS and cyclase activity. Different concentrations of 5-aminovaleric acid and 6-aminocaproic acid were added to LB medium containing GESS cells. Values represent mean ± SD of three independent experiments.

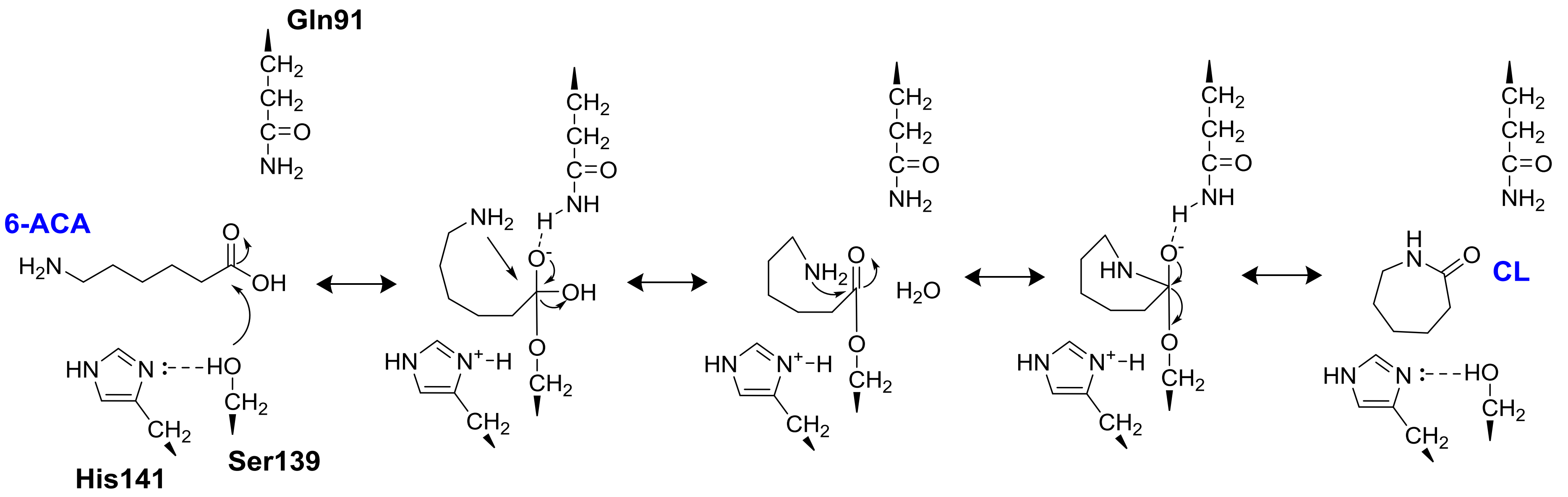

Supplementary Fig.19. Proposed mechanism of the reversible CF3HBD reaction. CF3HBD was proven to catalyze the interconversion between 6-ACA and ε-caprolactam in this study. A possible mechanism to this reversible reaction is that the S139-H141 pair act to generate a nucleophile to activate the carboxylic group of 6-ACA in the active site of CF3HBD. The reaction is facilitated by simultaneous proton transfer from Ser139 to His141 while the side chain of Q91 may form hydrogen bonds with the oxygen of the carbonyl group in 6-ACA.

Supplementary Table 1. Promoters and RBSs for combinatorial optimization of the CL-detective regulator circuits

Identifier		Sequence
Promoter	J23100*	TTGACGGCTAGCTCAGTCCTAGGTACAGTGCTAGC
	J23106	TTTACGGCTAGCTCAGTCCTAGGTATAGTGCTAGC
	J23114	TTTATGGCTAGCTCAGTCCTAGGTACAATGCTAGC
RBS	B0030	TCTAGAGATTAAAGAGGAGAAATACTAGATG
	B0034	TCTAGAGAAAGAGGAGAAATACTAGATG
	T7RBS	TCTAGAGAAGGAGATATACATATG

***Names adopted from the part-registry website, http://parts.igem.org/Promoters/Catalog/Anderson**

Supplementary Table 2. Determination of intracellular level of ε-caprolactam

**ε-Caprolactam (mM)**	**Intracellular ε-Caprolactam (μmole/cell-mg)**
0	ND
1	0.035 ± 0.001
10	0.129 ± 0.003
30	0.271 ± 0.041

Supplementary Table 3. Predicted ORFs in #3 positive clone that is isolated from metagenomic library

Name	Length	Description
Contig1_orf00001	155	Inorganic diphosphatase [*Citrobacter freundii* GTC 09479]
Contig1_orf00003	572	Protein YheS [*Citrobacter freundii* GTC 09479]
Contig1_orf00006	566	Malate dehydrogenase
Contig1_orf00009	257	**3-Hydroxybutyrate dehydrogenase**
Contig1_orf00010	465	Citrate transporter [*Citrobacter youngae* ATCC 29220] permease
Contig1_orf00012	295	3-Hydroxyacyl-CoA dehydrogenase [*Citrobacter freundii* GTC 09479]
Contig1_orf00013	402	Putative acetyl-CoA acetyltransferase [*Citrobacter freundii* GTC 09479]
Contig1_orf00015	219	Hypothetical protein WC1_02027 *[Citrobacter* sp. KTE30] succinyl-CoA:3-ketoacid-CoA transferase
Contig1_orf00016	262	3-Oxoacid CoA-transferase, A subunit [*Citrobacter youngae* ATCC 29220]
Contig1_orf00017	297	Hypothetical protein WC1_02025 [*Citrobacter* sp. KTE30]l LysR family transcriptional regulator
Contig1_orf00018	312	Hypothetical protein H262_01440 [*Citrobacter freundii* GTC 09479] ABC
Contig1_orf00019	329	D-Ala-D-Ala transporter subunit [*Citrobacter* sp. KTE30]
Contig1_orf00020	299	Hypothetical protein HMPREF9428_01324 [*Citrobacter freundii* 4_7_47CFAA] cytochrome C550
Contig1_orf00021	341	D-Ala-D-Ala transporter subunit [*Citrobacter* sp. KTE30] peptide ABC transporter permease
Contig1_orf00022	521	D-Ala-D-Ala transporter subunit [*Citrobacte*r sp. KTE30]
Contig1_orf00023	196	D-alanyl-D-alanine dipeptidase [*Citrobacter* sp. KTE30]
Contig1_orf00024	289	Hypothetical protein WC1_02018 [*Citrobacter* sp. KTE30] phosphosugar isomerases
Contig1_orf00025	57	No hit
Contig1_orf00027	648	Hypothetical protein H922_02186 [*Citrobacter freundii* GTC 09629] chemotaxis protein
Contig1_orf00029	424	Hypothetical protein WC1_02014 *[Citrobacter* sp. KTE30] 2 serine/threonine-protein kinase
Contig1_orf00030	173	Hypothetical protein WC1_02013 [*Citrobacter* sp. KTE30] 3 membrane protein
Contig1_orf00031	456	Hypothetical protein D186_02721 [*Citrobacter freundii* ATCC 8090 = MTCC 1658] glycoside hydrolase lipoprotein
Contig1_orf00033	443	Putative type I secretion membrane fusion protein *[Citrobacter freundii* GTC 09629]proteases lipase C
Contig1_orf00034	580	Hypothetical protein WC1_02009 [*Citrobacter* sp. KTE30] peptidase putative alkaline protease

Supplementary Table 4. Candidate ORFs in #3 positive clone for expression tests. 11 ORFs were subcloned into pET28a(+)

ORF No.	Gene name
1	Malate dehydrogenase
2	3-Hydroxybutyrate dehydrogenase
3	3-Hydroxyacyl-CoA dehydrogenase [*Citrobacter freundii* GTC 09479]
4	Putative acetyl-CoA acetyltransferase [*Citrobacter freundii* GTC 09479]
5	Succinyl-CoA:3-ketoacid-CoA transferase
6	D-Ala-D-Ala transporter subunit ,peptide ABC transporter permease
7	D-Alanyl-D-alanine dipeptidase [*Citrobacter* sp. KTE30]
8	Hypothetical protein WC1_02014 ,2 serine/threonine-protein kinase
9	Hypothetical protein D186_02721 , glycoside hydrolase lipoprotein
10	Putative type I secretion membrane fusion protein [*Citrobacter freundii* GTC 09629]proteases lipase C
11	Hypothetical protein WC1_02009 [*Citrobacter* sp. KTE30] peptidase putative alkaline protease

Supplementary Table 5. Primers used for the construction of new genetic circuits in this study

Construction	No.	Name	Sequence (5'-3')
CL-GESS$_{eGFP}$	1	nitR F	CGACAAGGAGGATGTCCATGGATGGAGCAATGGTCCACCA
	2	nitR R	AGAAGGAATAAGCTTGCCGGCCTTCATGATGATG
	3	PnitA F	GGGGCCGGCAAGCTTGCCGGCCTTCATGATGATG
	4	PnitA R	AAGGCCGGCAAGCTTGCCGGCCCCAGGT
	5	rrnBT F	ATGGACAAGCTGTACAAGTAAGCTTCTGTTTTGGCGGATGAGAGAAGA
	6	rrnBT R	AGCGGATAACAATTTCACACAGAAACAGCTATGACCATGATTACGCCAAGAGTTTGTAGAAACGCAAAAAG
	7	tL3 F	CCCGAATTCTTCTTCGTCTGTTTCTACTG
	8	tL3 R	CCCGAATTCAATGGCGATGACGCATCCTCA
	9	CL-GESS BB F	CTTTTTGCGTTTCTACAAACTCTTGGCGTAATCATGGTCATAGCTGTT
	10	CL-GESS BB R	ATACCAGTAGAAACAGACGAAGAATGGTGCACTCTCAGTACAATCTGC
CL-GESS$_{pBBRBB}$	11	pBBRBB F	CTGTTCCAGACTATACTAGAAAGAGTTTGTAGAAACGCAA
	12	pBBRBB R	CTGTTCCAGACTATACTAGAAAGAGTTTGTAGAAACGCAA
	13	pBBRBB BB F	TTGCGTTTCTACAAACTCTTTCTAGTATAGTCTGGAACAG
	14	pBBRBB BB R	CAGTAGAAACAGACGAAGAATCTAGTCCAACCTTTCATAG
CL-GESS$_{pSEVA234}$	15	pSEVA234 F	GAGAGCAAGCCCGTAGGGGGTTCTTCGTCTGTTTCTACTG
	16	pSEVA234 R	TCAGAGATTTTGAGACACAAAAGAGTTTGTAGAAACGCAA
	17	pSEVA234 BB F	TTGCGTTTCTACAAACTCTTTTGTGTCTCAAAATCTCTGA
	18	pSEVA234 BB R	CAGTAGAAACAGACGAAGAACCCCCTACGGGCTTGCTCTC
CL-GESS$_{sfGFP}$	19	sfGFP F	TCGCGGATACCGCGACAAGGAGATATACATATGAGCAAAGGTGAAGAACTGTTTACCGGC
	20	sfGFP R	ATCTTCTCTCATCCGCCAAAACAGAAGCTTATTTTTCGAACTGCGGATGGCTCCAC
	21	sfGFP BB F	GTGGAGCCATCCGCAGTTCGAAAAATAAGCTTCTGTTTTGGCGGATGAGAGAAGAT
	22	sfGFP BB R	GCCGGTAAACAGTTCTTCACCTTTGCTCATATGTATATCTCCTTGTCGCGGTATCCGCGA
CL-GESS$_{J23106/B0030-sfGFP}$	23	PB2 F	CACTATACCTAGGACTGAGCTAGCCGTAAAAAGCTTGCCGGCCTTCATGATGATGC
	24	PB2 R	CTAGCTCTAGAGATTAAAGAGGAGAAATACTAGATGGAGCAATGGTCCACCACGGCAATTCCCG
CL-GESS$_{J23114/B0030-sfGFP}$	25	PB3 F	GCATTGTACCTAGGACTGAGCTAGCCATAAAAAGCTTGCCGGCCTTCATGATGATGC
	26	PB3 R	TAGCTCTAGAGATTAAAGAGGAGAAATACTAGATGGAGCAATGGTCCACCACGGCAATTCCCG
CL-GESS$_{J23100/B0034-sfGFP}$	27	PB4 F	AGCACTGTACCTAGGACTGAGCTAGCCGTCAAAAGCTTGCCGGCCTTCATGATGATGC
	28	PB4 R	AGCTCTAGAGAAAGAGGAGAAATACTAGATGGAGCAATGGTCCACCACGGCAATTCCCG
CL-GESS$_{J23106/B0034-sfGFP}$	29	PB5 F	CACTATACCTAGGACTGAGCTAGCCGTAAAAAGCTTGCCGGCCTTCATGATGATGC
	30	PB5 R	CTAGCTCTAGAGAAAGAGGAGAAATACTAGATGGAGCAATGGTCCACCACGGCAATTCCCG
	31	PB6 F	CATTGTACCTAGGACTGAGCTAGCCATAAAAAGCTTGCCGGCCTTCATGATGATGC

CL-GESS$_{\text{J23114/B0034-sfGFP}}$	32	PB6 R	CTAGCTCTAGAGAAAGAGGAGAAATACTAGATGGAGCAATGGTCCACCACGGCAATTCCCG
CL-GESS$_{\text{J23100/T7RBS-sfGFP}}$	33	PB7 F	CTGTACCTAGGACTGAGCTAGCCGTCAAAAGCTTGCCGGCCTTCATGATGATGC
	34	PB7 R	TGCTAGCTCTAGAGAAGGAGATATACATATGGAGCAATGGTCCACCACGGCAATTCCCG
CL-GESS$_{\text{J23106/T7RBS-sfGFP}}$	35	PB8 F	CTATACCTAGGACTGAGCTAGCCGTAAAAAGCTTGCCGGCCTTCATGATGATGC
	36	PB8 R	TGCTAGCTCTAGAGAAGGAGATATACATATGGAGCAATGGTCCACCACGGCAATTCCCG
CL-GESS$_{\text{J23114/T7RBS-sfGFP}}$	37	PB9 F	TTGTACCTAGGACTGAGCTAGCCATAAAAAGCTTGCCGGCCTTCATGATGATGC
	38	PB9 R	TGCTAGCTCTAGAGAAGGAGATATACATATGGAGCAATGGTCCACCACGGCAATTCCCG
CL-GESS-sfGFP /PnitA truncation	39	PnitA200 F	ACCTAGGACTGAGCTAGCCGTCAAAAGCTTCCCCCAACCCTCTTGTTCACTGCACGGACGG
	40	PnitA300 F	ACCTAGGACTGAGCTAGCCGTCAAAAGCTTCACCATTCCCGGCTTGCTGATGCGCCCCATCCC
	41	PnitA R	TTCTTCACCTTTGCTCATATGTATATCTCCTTGTCGCGGTATCCGCGATCCTGATCGCCAG
	42	PnitABB F	CTGGCGATCAGGATCGCGGATACCGCGACAAGGAGATATACATATGAGCAAAGGTGAAGAA
	43	PnitA100 BB R	GTGTAGGAGGGGAAGGTGGCGATGAATTGAAAGCTTTTGACGGCTAGCTCAGTCCTAGGT
	44	PnitA200 BB R	CCGTCCGTGCAGTGAACAAGAGGGTTGGGGGAAGCTTTTGACGGCTAGCTCAGTCCTAGGT
	45	PnitA200 BB R	GGGATGGGGCGCATCAGCAAGCCGGGAATGGTGAAGCTTTTGACGGCTAGCTCAGTCCTAGGT
	46	PnitA200T 3’ F	AAGGAGATATACATATGAGCAAAG
	47	PnitA200T 3’/50bp R	CGTCTTGGTTTGCGTCAGTGTAGGA
	48	PnitA200T 3’/84bp R	GGTGGCGATGAATTGACTGACAGGC
	49	PnitA200T 3’/110bp R	CAGGATGCTTGACTGGGAGTCGCAA
	50	PnitA200T 3’/160bp R	CGTGCCTGGCCGTCCGTGCAGTGAA
	51	PnitA200T 5’/30bp F	GCCAGGCCAGGATGGGGGACTCCCGCTCCCCCTGCTTGCGACTCCCAGTC
	52	PnitA200T 5’/60bp F	CCTGCTTGCGACTCCCAGTCAAGCATCCTGCGCCTGTCAGTCAATTCATC
	53	PnitA200T 5’/80bp F	AAGCATCCTGCGCCTGTCAGTCAATTCATCGCCACCTTCCCCTCCTACACTGACGCAAAC
	54	PnitA200T 5’/100bp F	TCAATTCATCGCCACCTTCCCCTCCTACACTGACGCAAACCAAGACGACC
	55	PnitA200T 5’ R	AAGCTTTTGACGGCTAGCTCAGTCCTAGGTACAGTGCTAGCTACTAGAGA
pET-CF3HBD	56	pET-CF3HBD F	GGCCTGGTGCCGCGCGGCAGCCATATGAACTTAACGGGAAAAACCGCCC
	57	pET-CF3HBD R	GTGGTGGTGGTGGTGGTGCTCGAGTTATTGCGCTACCCAACCGCCATC
	58	pET-CF3HBD BB F	GATGGCGGTTGGGTAGCGCAATAACTCGAGCACCACCACCACCACCAC
	59	pET-CF3HBD BB R	GGGCGGTTTTTCCCGTTAAGTTCATATGGCTGCCGCGCGGCACCAGGCC

Supplementary Table 6. Primers used for NitR mutagenesis

	Name	Sequence (5'-3')
For error prone PCR of NitR	NitR F	GCGTCATCGCCATTGGTACCTTAGACAGGCATCGAATCTG
	NitR R	GAGATTAAAGAGGAGAAACCATGGAGCAATGGTCCACCAC
	Backbone F	GTGGTGGACCATTGCTCCATGGTTTCTCCTCTTTAATCTC
	Backbone R	CAGATTCGATGCCTGTCTAAGGTACCAATGGCGATGACGC
For site directed mutagenesis of NitR	L117F	CCACCCGCCCGTATCAGTTCTTGTTTGATCATGACTTC
	L117Y	GACTCCACCCGCCCGTATCAGTATTTGTTTGATCATGACTTCGAAC
	L117W	TCCACCCGCCCGTATCAGTGGTTGTTTGATCATGACTTC
	S133P	CTTTCTTTGCCGGGCCCCATCTTGCGCAAAC
	L117A	CCACCCGCCCGTATCAGGCGTTGTTTGATCATGACTT
	L117E	CCACCCGCCCGTATCAGGAGTTGTTTGATCATGACTT
	L117R	CCCGCCCGTATCAGCGGTTGTTTGATCATGAC
	L117Q	CCCGCCCGTATCAGCAGTTGTTTGATCATGAC

Supplementary Table 7. Primers used for site-directed mutagenesis of 3HBD

	Sequence (5'-3')
Q91A	TATCAATAACGCCGGGATCGCGCATGTTTCACCGATAGAA
Q91E	CAATAACGCCGGGATCGAGCATGTTTCACCGAT
S139A	GCATCATTAATATCGCTGCTGTTCATGGCCTGGTG
S139E	GGCGCATCATTAATATCGCTGAGGTTCATGGCCTGGTGGCTTC
V140A	AAGCCACCAGGCCGTGAGCAGAAGCGATATTAATG
V140E	TTGAAGCCACCAGGCCGTGCTCAGAAGCGATATTAATGAT
H141A	GCGCATCATTAATATCGCTTCTGTTGCTGGCCTGGTGGCT
H141E	GCGCATCATTAATATCGCTTCTGTTGAGGGCCTGGTGGCTT
K149A	CTGGTGGCTTCAAAAGAGGCATCTGCGTACGTAGCAGC
K149E	CTGGTGGCTTCAAAAGAGGAGTCTGCGTACGTAGCAGCG
Y152A	TCAAAAGAGAAATCTGCGGCCGTAGCAGCGAAGCACGG
Y152E	CAAAAGAGAAATCTGCGGAGGTAGCAGCGAAGCACGG
Q193A	GCCGCTGGTCCAGCAGGCGATCGATAAGCGTATT
Q193E	CGCTGGTCCAGCAGGAGATCGATAAGCGT
Q193E	ACGCTTATCGATCTCCTGCTGGACCAGCG
W184A	CGGCGTTAGCACCGCGCCGGGACACAGC
W184E	CGGCGTTAGCACCGCGCCGGGACACAGC
V190A	GATCTGCTGCTGGGCCAGCGGCGTTAG

Supplementary Table 8. Gene sequences of 3-Hydroxybutyrate dehydrogenase in this study

	Sequence (5’-3’)
DNA sequence	ATGAACTTAACGGGAAAAACCGCCCTGGTTACCGGCTCGACCAGCGGTATCGGATT<br>AGGTATCGCACAGGTGCTGGCGCAAGCTGGCGCCACCCTGATCCTCAACGGGTTT<br>GGTGATGTTGATGCCGCCAAAGACGCTGTTGCGCAGTATGGCAAAACGCCAGGCT<br>ATCATGGCGCGGATCTGAGCGATGAAGCGCAAATTGCCGACATGATGCGCTATGCA<br>GAGAGCGAATTCGGCGGTGTGGATATTCTTATCAATAACGCCGGGATCCAGCATGTT<br>TCACCGATAGAAACCTTCCCGGTTGATAAATGGAACGCGATTATCGCGATTAACCTC<br>TCCTCCGTTTTTCACACCACGCGTCTGGCGCTTCCCGGTATGCGCGCGCGAAACT<br>GGGGGCGCATCATTAATATCGCTTCTGTTCACGGCCTGGTGGCTTCAAAAGAGAAA<br>TCTGCGTACGTAGCAGCGAAGCACGGTGTGGTGGGATTAACCAAGACCATCGCGC<br>TGGAAACCGCGCAGACGGAAATTACCTGCAATGCGCTGTGTCCCGGCTGGGTGCT<br>AACGCCGCTGGTCCAGCAGCAGATCGATAAGCGTATTGCCGAACGCGCAGAGCCT<br>GAGGCTGCCCGTGACGCCCTGCTGGCTGAAAAGCAGCCGTCGCGCGAATTCGTTA<br>CCCCAGAGCAGTTAGGGAATCTTGCGTTATTCTTATGTTCAGACGGTGCGGCGCAA<br>GTGCGTGGCGTAGCGTGGAATATGGATGGCGGTTGGGTAGCGCAATAA
Amino acid sequence	MNLTGKTALVTGSTSGIGLGIAQVLAQAGATLILNGFGDVDAAKDAVAQYGKTPGYHGA<br>DLSDEAQIADMMRYAESEFGGVDILINNAGIQHVSPIETFPVDKWNAIIAINLSSVFHTTR<br>LALPGMRARNWGRIINIASVHGLVASKEKSAYVAAKHGVVGLTKTIALETAQTEITCNAL<br>CPGWVLTPLVQQQIDKRIAERAEPEAARDALLAEKQPSREFVTPEQLGNLALFLCSDG<br>AAQVRGVAWNMDGGWVAQZ
