# Supplementary material for: A synthetic microbial biosensor for high-throughput screening of lactam biocatalysts
Source: Nat Commun. 2018 Nov 29;9:5053. doi: 10.1038/s41467-018-07488-0 (PMC6265244; doi:10.1038/s41467-018-07488-0)
Supplement: Supplementary file 3 — Reporting Summary [file 41467_2018_7488_MOESM3_ESM.pdf]

natureresearch

Corresponding author(s): Seung-Goo Lee

Reporting Summary

Nature Research wishes to improve the reproducibility of the work that we publish. This form provides structure for consistency and transparency in reporting. For further information on Nature Research policies, see Authors & Referees and the Editorial Policy Checklist.

Statistical parameters

When statistical analyses are reported, confirm that the following items are present in the relevant location (e.g. figure legend, table legend, main text, or Methods section).

n/a	Confirmed	
☐	☒	The exact sample size (n) for each experimental group/condition, given as a discrete number and unit of measurement
☐	☒	An indication of whether measurements were taken from distinct samples or whether the same sample was measured repeatedly
☒	☐	The statistical test(s) used AND whether they are one- or two-sided<br>*Only common tests should be described solely by name; describe more complex techniques in the Methods section.*
☒	☐	A description of all covariates tested
☐	☒	A description of any assumptions or corrections, such as tests of normality and adjustment for multiple comparisons
☐	☒	A full description of the statistics including central tendency (e.g. means) or other basic estimates (e.g. regression coefficient) AND variation (e.g. standard deviation) or associated estimates of uncertainty (e.g. confidence intervals)
☒	☐	For null hypothesis testing, the test statistic (e.g. F, t, r) with confidence intervals, effect sizes, degrees of freedom and P value noted<br>*Give P values as exact values whenever suitable.*
☒	☐	For Bayesian analysis, information on the choice of priors and Markov chain Monte Carlo settings
☐	☒	For hierarchical and complex designs, identification of the appropriate level for tests and full reporting of outcomes
☒	☐	Estimates of effect sizes (e.g. Cohen's d, Pearson's r), indicating how they were calculated
☐	☒	Clearly defined error bars<br>*State explicitly what error bars represent (e.g. SD, SE, CI)*

Our web collection on statistics for biologists may be useful.

Software and code

Policy information about availability of computer code

Data collection	The data were acquired using FACSDiva 7.0 for flow cytometry, OpenLab CDS ChemStation Edition C.01.05 for LC & LC/MS systems, GC ChemStation Software B.04.03, PerkinElmer 2030 Workstation, NIS Elements 4.20 for microscopy.
Data analysis	The data were analyzed using SigmaPlot 12, FlowJo V10, ImageJ 1.51k

For manuscripts utilizing custom algorithms or software that are central to the research but not yet described in published literature, software must be made available to editors/reviewers upon request. We strongly encourage code deposition in a community repository (e.g. GitHub). See the Nature Research guidelines for submitting code & software for further information.

Data

Policy information about availability of data

All manuscripts must include a data availability statement. This statement should provide the following information, where applicable:

- Accession codes, unique identifiers, or web links for publicly available datasets
- A list of figures that have associated raw data
- A description of any restrictions on data availability

The refined models of CF3HBD have been deposited in the Protein Data Bank (http://www.rcsb.org/) with PDB code 5YSS. All data that support the findings of this study are included in this article and in Supplementary Information. They are available from the corresponding author upon reasonable request.

April 2018

Field-specific reporting

Please select the best fit for your research. If you are not sure, read the appropriate sections before making your selection.

☒ Life sciences ☐ Behavioural & social sciences ☐ Ecological, evolutionary & environmental sciences

For a reference copy of the document with all sections, see nature.com/authors/policies/ReportingSummary-flat.pdf

Life sciences study design

All studies must disclose on these points even when the disclosure is negative.

Sample size	The sample size was chosen based on the author's experience and previously published peer-reviewed papers. Sufficient sample size was employed in each analysis.
Data exclusions	No data was excluded from the analysis.
Replication	All experiments were reproduced to verify the reproducibility of the experimental findings. All replication attempts were successful.
Randomization	The sample analysis was performed randomly using flow cytometry or microscopy. No data was excluded from the analysis.
Blinding	Data collection and analysis was done 'blind'. No data was excluded.

Reporting for specific materials, systems and methods

Materials & experimental systems

n/a	Involved in the study
☒	☐ Unique biological materials
☒	☐ Antibodies
☒	☐ Eukaryotic cell lines
☒	☐ Palaeontology
☒	☐ Animals and other organisms
☒	☐ Human research participants

Methods

n/a	Involved in the study
☒	☐ ChIP-seq
☐	☒ Flow cytometry
☒	☐ MRI-based neuroimaging

Flow Cytometry

Plots

Confirm that:

☒ The axis labels state the marker and fluorochrome used (e.g. CD4-FITC).

☒ The axis scales are clearly visible. Include numbers along axes only for bottom left plot of group (a 'group' is an analysis of identical markers).

☒ All plots are contour plots with outliers or pseudocolor plots.

☒ A numerical value for number of cells or percentage (with statistics) is provided.

Methodology

Sample preparation	E. coli EPI300 cells as bacterial sample harboring the metagenomic library were electrophoretically transformed with the CL-GESS plasmid and were grown on LB agar containing 50 mM 6-aminocaproic acid, 100 µg/ml ampicillin, and 34 µg/ml chloramphenicol at 37°C for 14 h.
Instrument	FACSAria III instrument (BD Biosciences, Franklin Lakes, NJ, USA)
Software	Data were acquired using FACSDiva 7.0 software and analyzed with FlowJo V10.
Cell population abundance	A blue laser (488 nm) and bandpass filter (530/30 nm) were used to analyze fluorescence intensity of the mutant library. Approximately 20,000 cells with high fluorescence intensity (top 0.4%) in 5.6 × 10^6 cells were collected
Gating strategy	A blue laser (488 nm) and bandpass filter (530/30 nm) were used to analyze fluorescence intensity of the mutant library.

Gating strategy

Approximately 20,000 cells with high fluorescence intensity (top 0.4%) in 5.6 × 10^6 cells were collected and were recovered in LB medium at 37°C for 16 h. In the second round of screening, false-positive cells showing high fluorescence intensity in the absence of ε-caprolactam were removed by sorting non-fluorescent cells. Approximately 1.5 × 10^5 (bottom 3%) non-fluorescent cells in 3 × 10^6 negatively sorted cells were collected and were grown in LB medium.

☒ Tick this box to confirm that a figure exemplifying the gating strategy is provided in the Supplementary Information.
